# Roles of Prostaglandins and Hydrogen Sulfide in an Outflow Model of the Porcine Ocular Anterior Segment Ex Vivo

**DOI:** 10.3390/ph17101262

**Published:** 2024-09-25

**Authors:** Jenaye Robinson, Leah Bush, Anthonia Okolie, Fatima Muili, Sunny Ohia, Catherine Opere, Ya Fatou Njie Mbye

**Affiliations:** 1 Department of Pharmaceutical Sciences, College of Pharmacy and Health Sciences, Texas Southern University, Houston, TX 77004, USA; jenayerobinson@yahoo.com (J.R.); lemwjb@gmail.com (L.B.); a.okolie9552@student.tsu.edu (A.O.); f.muili1867@student.tsu.edu (F.M.); sunny.ohia@tsu.edu (S.O.); 2 Department of Pharmacy Sciences, School of Pharmacy and Health Professions, Creighton University, Omaha, NE 68178, USA; catherineopere@creighton.edu

**Keywords:** aqueous humor outflow, hydrogen sulfide, prostaglandins, trabecular meshwork, perfusion pressure

## Abstract

Background: Hydrogen sulfide (H_2_S)-releasing compounds can reduce intraocular pressure in normotensive rabbits by increasing aqueous humor (AH) outflow through the trabecular meshwork. In the present study, we investigated the contribution of endogenous H_2_S and the role of intramurally generated prostaglandins in the observed increase in AH outflow facility in an ex vivo porcine ocular anterior segment model. Material and Methods: Porcine ocular anterior segment explants were perfused with Dulbecco’s Modified Eagle’s Medium maintained at 37 °C and gassed with 5% CO_2_ and 95% air under an elevated pressure of 15 mmHg for four hours. Perfusates from the anterior segment explants were collected and immediately assayed for their H_2_S and prostaglandin E_2_ content. Results: Elevating perfusion pressure from 7.35 to 15 mm Hg significantly (*p* < 0.001) increased H_2_S concentration in the perfusate from 0.4 ± 0.1 to 67.6 ± 3.6 nM/µg protein. In the presence of an inhibitor of cystathionine ß-synthase/cystathionine γ-lyase, aminooxyacetic acid (AOAA, 30 µM), or an inhibitor of 3-mercaptopyruvate sulfurtransferase, α-ketobutyric acid (KBA, 1 mM), the effects of elevated pressure on H_2_S levels in the perfusate was significant (*p* < 0.001). Furthermore, flurbiprofen (30 µM) and indomethacin (10 µM) attenuated the elevated pressure-induced increase in H_2_S levels in the perfusate. Interestingly, elevating perfusion pressure had no significant effect on PGE_2_ concentrations in the perfusate. While the inhibition of H_2_S biosynthesis by AOAA or KBA did not affect PGE_2_ levels in perfusate, flurbiprofen (30 µM) caused a significant (*p* < 0.05) decrease in the concentration of PGE_2_ under conditions of elevated perfusion pressure. Conclusions: We conclude that the elevated perfusion pressure-induced increase in H_2_S concentrations depends upon the endogenous biosynthesis of H_2_S and intramurally produced prostaglandins in the porcine anterior segment explants. While the concentration of PGE_2_ in the perfusate under elevated perfusion pressure was unaffected by pretreatment with inhibitors of H_2_S biosynthesis, it was reduced in the presence of an inhibitor of cyclooxygenase.

## 1. Introduction

For centuries, hydrogen sulfide (H_2_S) was known as a toxic gaseous compound until recently when new evidence emerged to support its biological role in mammalian organs and tissues [1,2,3]. Indeed, H_2_S has been reported to have important physiological and pharmacological actions similar to those of other gaseous transmitters such as nitric oxide and carbon monoxide [4]. In the cytosol, H_2_S biosynthesis occurs through its substrate, L-cysteine, in a reaction that is catalyzed by the pyridoxal-5-phosphate (P5P)-dependent enzymes, cystathionine β-synthase (CBS) and cystathionine γ-lyase (CSE) [1,5]. In addition to the cytosol, L-cysteine in the mitochondria can produce H_2_S in the presence of cysteine aminotransferase (CAT) and 3-mercaptopyruvate sulfur transferase (3-MST) in a reaction that is dependent upon calcium [6,7]. Recently, a fourth enzyme, D-amino acid oxidase (DAO), has been shown to catalyze the formation of H_2_S from D-cysteine in mammalian tissues [8]. In the eye, CBS, CSE, 3-MST, and DAO are involved in the biosynthesis of H_2_S and are highly expressed in tissues such as the iris, ciliary body, and retina [9,10]. It is well established that intraocular pressure (IOP) is maintained by a delicate balance between aqueous humor production in the ciliary body and its drainage via two outflow pathways. Approximately 75% of aqueous humor outflow occurs through the trabecular meshwork (TM) and Schlemm’s canal [11]. There is evidence that a dysfunction of the TM is one of the primary contributory factors that leads to elevated IOP and subsequent retinal damage and vision loss [11]. Compounds that lower IOP exert their pharmacological effects through various mechanisms, one of which is by modulating TM outflow facility [12]. Interestingly, H_2_S (using H_2_S-releasing compounds as gas donors) has been reported to lower IOP and increase trabecular meshwork outflow facility in experimental animal models [13,14]. In 2017, Huang and coworkers reported that endogenous levels of H_2_S and the expression of enzymes responsible for the biosynthesis of this gas (CBS, CSE, and 3MST) are significantly reduced in retinal ganglion cells in a rat model of ocular hypertension [15]. This finding suggests a possible involvement of an endogenous pathway leading to the biosynthesis of H_2_S in the regulation of IOP and maintenance of aqueous humor dynamics.

Prostaglandins are pro-inflammatory mediators that have been reported to be present at high concentrations in the aqueous humor of glaucomatous humans and experimental animal models [16,17]. Evidence from studies performed in our laboratory demonstrates that endogenous prostaglandins are involved in the pharmacological actions of H_2_S-releasing compounds in the eye [13,14,18,19]. Taken together, evidence from our laboratory and the published work of other investigators support a potential role for prostaglandins in the pharmacological actions of H_2_S on intraocular pressure. The demonstration of a possible interaction between these modulators in the TM outflow pathway may prove useful in understanding their role in aqueous humor dynamics. In the present study, we sought to determine whether a relationship exists between endogenous concentrations of H_2_S and prostaglandins in an ex vivo porcine anterior segment explant model designed to study TM outflow facility. Consequently, the aim of the present study was two-fold: (1) to determine the role of the endogenous pathway for the biosynthesis of H_2_S in a porcine ex vivo model of TM outflow facility under conditions of elevated perfusion pressure and (2) to investigate the interaction between endogenous prostaglandins and H_2_S in the pathway leading to the outflow of aqueous humor under conditions of elevated perfusion pressure.

## 2. Results

### 2.1. Effect of Elevated Perfusion Pressure on Hydrogen Sulfide Levels in TM Outflow Pathway

**Figure 1:**
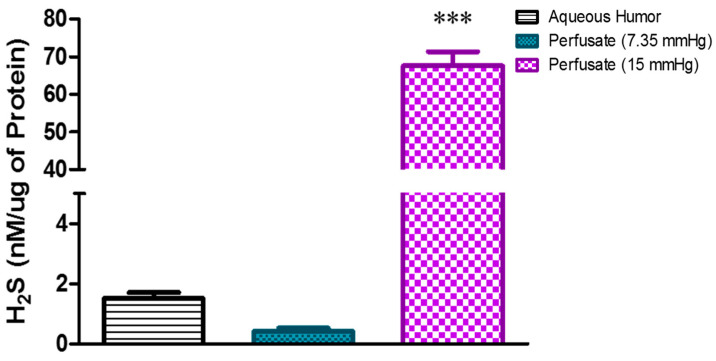
Effect of different perfusion pressures on hydrogen sulfide (H_2_S) concentrations in the perfusate of porcine anterior segment explant trabecular meshwork outflow facility, ex vivo. Untreated porcine anterior segment explants were mounted in perfusion culture chambers perfused with DMEM for four hours at 7.35 mm Hg (normal) or 15 mm Hg (elevated). Porcine aqueous humor H_2_S levels were measured as a reference point for comparison. Results are expressed as mean ± standard error. N = 12. *** significant difference between 7.35 and 15 mm Hg perfusate *p* < 0.001.

**Figure 2:**
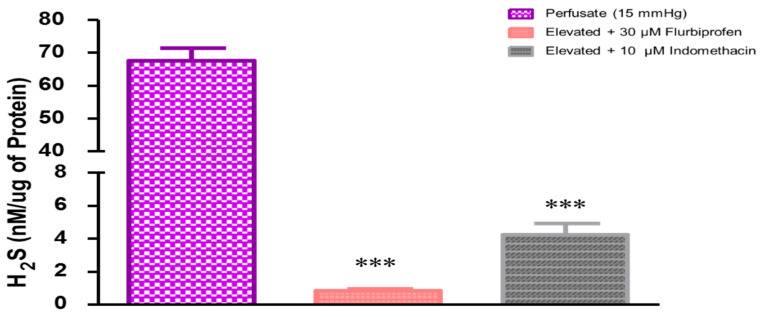
Effects of inhibitors of cyclooxygenase on hydrogen sulfide (H_2_S) concentrations in porcine anterior segment explant trabecular meshwork outflow facility, ex vivo. Control (at elevated perfusion pressure of 15 mm Hg) and in the presence of flurbiprofen (10 µM) or indomethacin (10 µM) for four hours. Results are expressed as mean ± standard error. N = 12. *** significant difference between flurbiprofen- and indomethacin-treated eyes and 15 mm Hg perfusate *p* < 0.001.

**Figure 3:**
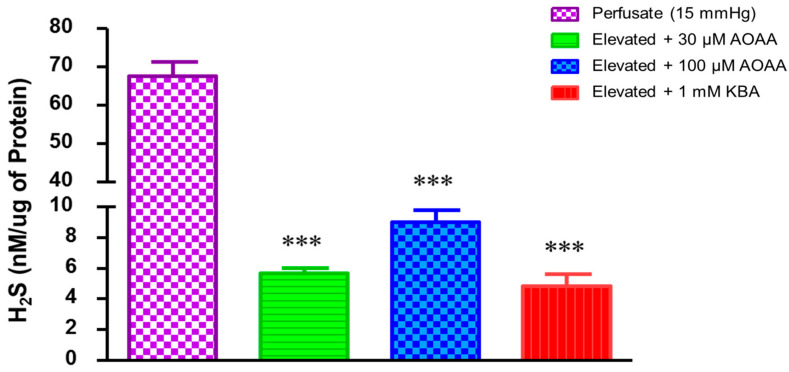
Effects of inhibitors of enzymes responsible for the biosynthesis of hydrogen sulfide (H_2_S) on H_2_S concentrations in porcine anterior segment explant trabecular meshwork outflow facility, ex vivo. Control (at elevated perfusion pressure of 15 mm Hg) and in the presence of Aminooxyacetic acid (AOAA) (30–100 µM) or α-Ketobutyric acid (KBA) (1 mM) for four hours. Results are expressed as mean ± standard error. N = 12. *** significant difference AOAA- or KBA-treated eyes and 15 mm Hg perfusate *p* < 0.001.

To assess the effects of elevated perfusion pressure on endogenous H_2_S production in porcine anterior segment explants, an experiment was designed to measure TM outflow facility with the perfusion pressure raised from 7.35 mm Hg to 15 mm Hg. The baseline concentration of H_2_S in the perfusate at 7.35 mm Hg was 0.41 ± 0.1 nM/µg protein, which increased significantly (*p* < 0.001) after the perfusion pressure was elevated to 15 mm Hg, yielding a concentration of 67.6 ± 3.7 nM/µg protein (*p* < 0.001) (Figure 1). We found the basal concentration of H_2_S in the porcine aqueous humor (used as a reference point) to be 1.5 ± 0.2 nM/µg protein. In a second set of experiments, we evaluated whether the perfusion pressure-induced increase in H_2_S concentrations involved enzymes responsible for the biosynthesis of H_2_S. Anterior segment explants were perfused with an inhibitor of CBS/CSE (AOAA, 30–100 µM; [20]) or an inhibitor of 3-MST (KBA, 1 mM; [21]) for four hours before the measurement of concentrations of H_2_S in the perfusate. In the presence of AOAA and KBA, the elevated perfusion pressure-induced increase in H_2_S levels was significantly (*p* < 0.001) attenuated (Figure 2). At a perfusion pressure of 15 mm Hg, the H_2_S concentration in the perfusate (67.6 ± 3.7 nM/µg protein) was significantly (*p* < 0.001) lowered to 5.7 ± 0.3 nM/µg protein and 9.0 ± 0.7 nM/µg protein following treatment with AOAA (30 µM and 100 µM, respectively). In the presence of KBA (1 mM), H_2_S levels were significantly (*p* < 0.001) lowered to 4.9 ± 0.8 nM/µg protein in the perfusate when compared to untreated controls (67.6 ± 3.7 nM/µg protein) (Figure 3).

### 2.2. Role of Prostaglandins in Elevated Perfusion Pressure-Induced Increase in Hydrogen Sulfide Levels in TM Outflow Pathways

Evidence from our laboratory and that reported by other investigators shows that H_2_S can stimulate the biosynthesis of prostaglandins and that prostaglandins may play a modulatory role on the pharmacological actions of H_2_S [13,18,19,20,21,22,23]. In the present study, we evaluated the effect of intramurally generated prostaglandins on the endogenous levels of H_2_S under conditions of elevated perfusion pressure in a porcine anterior segment explant model designed to measure TM outflow facility, ex vivo. Pretreatment with the non-selective COX inhibitor flurbiprofen (30 µM) or indomethacin (10 µM) significantly (*p* < 0.001) lowered the levels of endogenous H_2_S (Figure 3). In the presence of flurbiprofen or indomethacin, the elevated H_2_S levels due to increased perfusion pressure were 0.8 ± 0.1 and 4.2 ± 0.7 nM/µg protein, respectively (Figure 3).

### 2.3. Role of Hydrogen Sulfide in Elevated Perfusion Pressure on Prostaglandin Levels in TM Outflow Pathways

**Figure 4:**
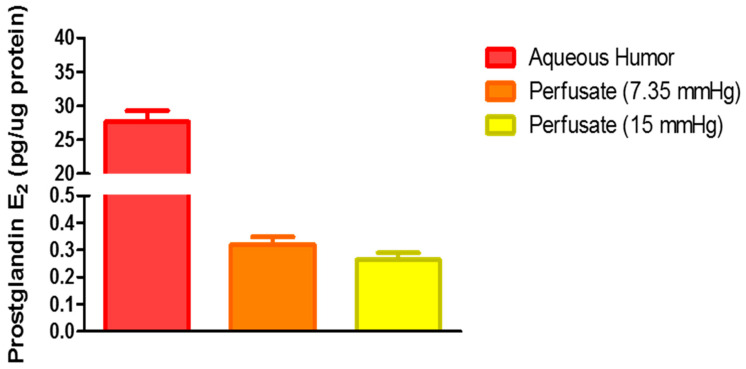
Effect of different perfusion pressures on prostaglandin E_2_ (PGE_2_) concentrations in the perfusate of porcine anterior segment explant trabecular meshwork outflow facility, ex vivo. Untreated porcine anterior segments were mounted in perfusion culture chambers perfused with DMEM for four hours at 7.35 mm Hg (normal) or 15 mm Hg (elevated). Porcine aqueous humor PGE_2_ levels were measured as a reference point for comparison. Results are expressed as mean ± standard error. N = 6.

In another set of experiments, we investigated whether elevated perfusion pressure-induced increase in H_2_S concentrations involved pathways leading to the biosynthesis of prostanoids such as prostaglandin E_2_ (PGE_2_). As shown in Figure 4, the concentration of PGE_2_ in the aqueous humor was 27.67 ± 1.59 pg/µg protein. Furthermore, there was no significant difference (*p* > 0.05) in PGE_2_ levels in the perfusate at normal and elevated perfusion pressure. The inhibition of enzymes responsible for the biosynthesis of endogenous H_2_S (CBS/CSE) by AOAA (30 µM) or 3-MST by KBA (1 mM) did not affect PGE_2_ levels. However, the non-specific COX inhibitor, flurbiprofen, significantly (*p* < 0.05) lowered PGE_2_ levels to 0.18 ± 0.01 pg/µg protein when compared to the untreated 15 mm Hg perfusate (0.26 ± 0.03 pg/µg protein).

## 3. Discussion

The presence of an endogenous pathway for the biosynthesis of H_2_S in ocular tissues indicates a potential physiological role for H_2_S in the eye, especially in the maintenance of IOP and aqueous humor dynamics [6,24]. There is evidence that H_2_S-releasing compounds can lower IOP in normal and glaucomatous animal models and that these ocular hypotensive actions are mediated by the endogenous biosynthesis of H_2_S [13]. We also have evidence that under normal perfusion pressure, H_2_S (administered as NaHS) and a substrate for the biosynthesis of this gas (L-cysteine) can increase aqueous humor outflow facility in porcine anterior segment explants, ex vivo, a response mediated at least in part by endogenously produced H_2_S, K_ATP_ channels and adenylyl cyclase [14]. The concept of doubling perfusion pressures as a marker of elevated intraocular pressures has been extensively reported by Acott and his coworkers [25,26,27]. Consequently, we used 15 mmHg to reflect the doubling of our normal value of 7.35 mm Hg. The rationale for studying trabecular meshwork (TM) outflow facility under conditions of elevated pressure is designed to mimic the pathophysiology of glaucoma. In the present study, we observed that there is a significant increase in H_2_S concentrations in the perfusate of porcine anterior segment explants TM outflow facility model following an increase in pressure from 7.35 mm Hg to 15 mm Hg. This observed increase in endogenous H_2_S levels may play a vital role in regulating TM outflow facility and subsequently IOP. Our findings are similar to those reported by Huang et al. [15], which showed that there was a three-fold increase in the expression of the 3-MST enzyme in the rat retina of an animal model of glaucoma, indicating a possible increase in the endogenous biosynthesis of H_2_S under conditions of elevated IOP [15]. Although there are no studies reported in the literature about changes in concentrations of H_2_S in perfusate from an anterior segment explant model designed for measurement of TM outflow facility, there is evidence of a significant increase in nitric oxide levels in perfusate of human anterior segments explants following changes in perfusion pressure from 10 mm Hg to 25 mm Hg [28]. Interestingly, in a previous study, we found that nitric oxide donors can increase H_2_S levels in isolated ocular tissues [29]. It is, therefore, tempting to speculate that the increased concentrations of H_2_S observed in response to elevated perfusion pressure in the porcine anterior segment explants may be secondary to an increase in nitric oxide in the TM outflow channels [28]. In summary, our results demonstrate that the concentration of H_2_S in the perfusate of porcine ocular anterior segment explants is dependent upon perfusion pressure. The enzymatic pathway for the biosynthesis of H_2_S from L-cysteine by CBS, CSE, and 3-MST is well documented [6,7,30]. However, it is pertinent to note that the endogenous production of H_2_S can also occur independently of these enzymes from processes such as glutathione reduction or the reduction in sulfur and polysulfides [31]. Based on evidence from previous studies, it was of interest to determine if an enzymatic conversion of L-cysteine to H_2_S was involved in the perfusion pressure-induced increase in H_2_S levels. We found that the CBS/CSE inhibitor, AOAA, and the 3-MST inhibitor, KBA, abolished the increase in H_2_S levels due to the increased perfusion pressure. These observations strongly suggest that the perfusion pressure-induced increase in the endogenous biosynthesis of H_2_S accounts, at least in part, for the observed augmented gas concentrations in the perfusate.

To elucidate a possible mechanism of perfusion pressure-induced increase in H_2_S levels in the perfusate of porcine anterior segment explants, we considered the potential involvement of prostaglandins as mediators of this response. In the present study, we found that the inhibition of prostaglandin biosynthesis by flurbiprofen or indomethacin elicited a significant (*p* < 0.001) attenuation of H_2_S levels induced by the increased perfusion pressure. Based on this observation, it appears that the increased level of H_2_S in the perfusate in response to elevated perfusion pressure in the anterior segment explants is due, at least in part, to the biosynthesis of prostaglandins. While there is ample evidence in the literature of prostaglandins being involved in the pharmacological actions of H_2_S [13,22,23,32], this is the first report of these eicosanoids being involved in the pathway leading to the production of H_2_S. Be that as it may, the present observation that changes in the biosynthesis of prostaglandins can alter the concentrations of H_2_S in the perfusate of porcine anterior segment explants is interesting and merits further investigation.

Prostaglandin analogs are widely used as IOP-lowering compounds in experimental animals and as drugs for the treatment of glaucoma [33,34]. Although prostaglandins lower IOP by increasing outflow through the unconventional (uveoscleral) pathway, there is evidence that suggests that they may also mediate the outflow of aqueous humor via the TM outflow channels [33,35]. In the present study, we sought to determine the relationship between changes in the perfusion pressure of porcine anterior segment explants and PGE_2_ levels in the perfusate. We now report that increasing perfusion pressure from 7.35 mm Hg to 15 mm Hg had no significant effect on PGE_2_ concentrations in the perfusate. A study by Maihoefner and colleagues reported a decrease in PGE_2_ levels in the aqueous humor of patients with primary open-angle glaucoma (POAG) and complete loss of COX-2 expression in the ciliary body when compared to healthy eyes [36]. These observations led these authors to conclude that the loss of COX-2 expression in the ciliary body and decreased PGE_2_ concentrations in the aqueous humor could be linked to the incidence of POAG [36]. In contrast, in the present study, we found that increasing ocular perfusion pressure did not affect the concentrations of PGE_2_ in the perfusate indicating that no such relationship exists between perfusion pressure and eicosanoid levels in the TM outflow channels in the porcine anterior segment explants, ex vivo.

There is evidence of the ability of H_2_S to regulate prostaglandin levels in mammalian tissues and organs. For instance, while Na_2_S and L-cysteine can inhibit PGE_2_ production in the rat hypothalamus, it was found to stimulate the expression of prostaglandin metabolizing enzymes in human uterine tissue [37,38]. In the present study, we investigated the possibility that increasing perfusion pressure and subsequent increases in H_2_S concentrations in the perfusate of porcine anterior segment explants can alter PGE_2_ levels in this system. There is evidence that endogenously formed H_2_S can regulate COX-2 and PGE_2_ expression in an acute lung injury mouse model [39]. Surprisingly, we observed that inhibition of enzymes responsible for the biosynthesis of H_2_S by AOAA or KBA did not affect PGE_2_ levels. As a positive control, we found that the cyclooxygenase inhibitor, flurbiprofen, significantly lowered the levels of PGE_2_ in the perfusate. A possible explanation for the lack of effect of inhibitors of the biosynthesis of H_2_S on PGE_2_ levels in the perfusate could be because the synthesis of new gas may not have a direct action on the concentration of already formed PGE_2_ detected in the perfusate. Indeed, the lack of an expected change in the concentration of PGE_2_ in the perfusate under conditions of elevated perfusion in the anterior segment explants appears to support our explanation. Another plausible explanation could be that the concentrations of newly synthesized gas may be too small to affect the biosynthetic pathway, leading to the biosynthesis of PGE_2_ in the explants.

A possible practical application of the findings of the present study is in understanding the role of H_2_S (both exogenous and endogenous) in the regulation of outflow facility in the TM. Data from our previous work demonstrate the ability of H_2_S-releasing compounds to increase outflow facility, a response that was dependent on endogenous biosynthesis of the gas, adenylyl cyclase activity, and K^+^-ATP channels [14]. In the present study, we found that the elevation of perfusion pressure increased the production of endogenous H_2_S, suggesting a role for this gas in the regulation of aqueous humor dynamics during normal and increased intraocular pressure observed during glaucoma.

In summary, the effect of changes in perfusion pressure on eicosanoid levels in the absence and/or presence of H_2_S in the perfusate of porcine anterior segment explant model of TM outflow channels is of interest and should be thoroughly investigated.

## 4. Materials and Methods

### 4.1. Chemicals

The substrate for H_2_S synthesis (L-cysteine), CBS/CSE inhibitor (aminooxyacetic acid (AOAA)), 3-MST inhibitor (alpha-ketobutyric acid (KBA), and indomethacin were procured from Sigma-Aldrich (St. Louis, MO, USA). Flurbiprofen was purchased from Cayman Chemical (Ann Arbor, MI, USA). All test agents were freshly prepared immediately before use on the day of the study. Stock solutions of flurbiprofen were dissolved in 70% ethanol. All other test compounds were dissolved in distilled water. Fresh porcine aqueous humor was collected for the measurement of basal H_2_S concentrations. The number of explants used for each experiment ranged from 8 to 12. Parallel measurements were made for control explants and those exposed to drugs/chemicals.

### 4.2. Porcine Anterior Segment Perfusion Model, Ex Vivo

**Figure 5:**
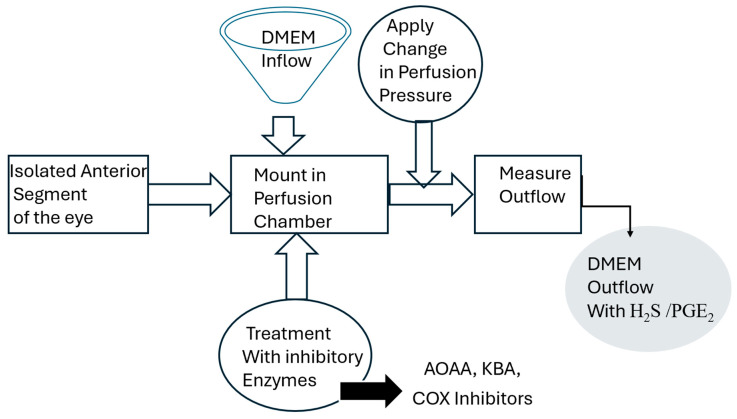
Experimental design.

Fresh porcine eyes obtained from Vision Tech (Dallas, TX, USA) were transported to the laboratory in saline on ice within 24 h following enucleation. The methodology de-scribed by us for setting up the perfused porcine ocular anterior segment organ culture model was employed for these experiments [14,40]. Briefly, porcine ocular anterior segment explants were carefully dissected by removing the iris and ciliary muscle and leaving only the trabecular meshwork, cornea, and small (5 mm) ring of sclera. Explants were then mounted onto perfusion culture chambers perfused with DMEM (supplemented with penicillin and streptomycin) using a constant perfusion headset at a height of 10 or 20 cm for a constant pressure of 7.35 and 15 mm Hg, respectively [41]. Explants were treated with enzyme inhibitors of the biosynthesis of H2S (AOAA, 30–100 µM or KBA, 1 mM) or inhibitors of cyclooxygenase (flurbiprofen, 10 µM or indomethacin, 10 µM) for four hours. At the end of the study, outflow was clamped off, and the perfusate was carefully collected from the anterior segment using a syringe. (Figure 5).

The isolated anterior segment of the eye includes the cornea, sclera, and the trabecular meshwork.

DMEM—Dulbecco’s Modified Eagle’s Medium; AOAA—aminooxyacetic acid; KBA—α-ketobutyric acid; COX inhibitors—flurbiprofen, and indomethacin.

### 4.3. Measurement of H_2_S and Prostaglandin E_2_ Concentrations

The concentration of H_2_S in the media perfusing the explants in the chamber (i.e., perfusate) was measured by the Methylene Blue assay method as described by several investigators [29,42,43] and prostaglandin E_2_ (PGE_2_) was measured using the Enzyme Immunoassay (EIA) kit following the manufacturer’s instructions (Cayman Chemical, Ann Arbor, MI, USA). After four hours, perfusates were collected, combined with 1% EDTA, 1% zinc acetate, and borate buffer (pH 11). The mixture was incubated in a 37 °C water bath for 30 min. Thereafter, N,N dimethyl p-phenylendiamine (20 mM in 7.2 M HCl) and FeCl_3_ (300 mM) were added and incubated at 37 °C with shaking for 10 min. The resulting solution absorbance was measured at 670 nm with a spectrophotometer (Bio Tek Microplate Instrumentation, U.S. Biotek Laboratories, Shoreline, WA, USA). The concentrations of H_2_S in perfusate was calculated based on a standard curve of sodium hydrosulfide (NaHS, 0–750 µM). H_2_S concentrations were expressed as nM/µg protein.

In assays to measure PGE_2_ concentrations, the perfusate was pretreated with indomethacin to prevent the further biosynthesis of eicosanoids during the assay period. PGE_2_ levels were measured using a Prostaglandin E_2_ Enzyme Immunoassay (EIA) kit following the manufacturer’s instructions (Cayman Chemical, Ann Arbor, MI, USA). PGE_2_ levels are expressed as pg/µg of protein.

### 4.4. Protein Measurement

Protein content in all samples from H_2_S and PGE_2_ assays was measured using the Bradford Method (Cayman Chemical, Ann Arbor, MI, USA) [44].

### 4.5. Statistics

Data are represented as the mean ± standard error and were analyzed using a GraphPad software (10.2.3 (403)). Increase in outflow facility compared to vehicle was considered statistically significant if *p* < 0.05 as analyzed by Student’s *t*-test or one-way ANOVA with Tukey’s post-test. Changes in endogenous levels of H_2_S or PGE_2_ was deemed statistically significant if *p* < 0.05 using Student’s *t*-test.

## 5. Conclusions

We conclude that in the perfused porcine ocular anterior segment model of TM outflow facility, the elevation of perfusion pressure elicits an increase in H_2_S levels but not that of PGE_2_. Furthermore, the perfusion pressure-induced changes in H_2_S levels are dependent upon the biosynthesis of endogenous H_2_S and prostaglandins. Further studies are needed to elucidate the mechanism/s that underlie the observed changes in the concentrations of H_2_S and prostaglandins in the perfusate of anterior segment explants under elevated perfusion pressure. It remains to be determined whether these observations ex vivo have any correlation with the outflow of aqueous humor from TM pathways during the regulation of aqueous humor dynamics and IOP in vivo.

## Data Availability

The data have been presented in the main text.

## References

[1] Kimura H. (2011). Hydrogen sulfide: Its production, release and functions. Amino Acids.

[2] Kimura H. (2021). Hydrogen Sulfide (H_2_S) and Polysulfide (H_2_S_n_) Signaling: The First 25 Years. Biomolecules.

[3] Tan B.H., Wong P.T., Bian J.S. (2010). Hydrogen sulfide: A novel signaling molecule in the central nervous system. Neurochem. Int..

[4] Li L., Rossoni G., Sparatore A., Lee L.C., Del Soldato P., Moore P.K. (2007). Anti-inflammatory and gastrointestinal effects of a novel diclofenac derivative. Free Radic. Biol. Med..

[5] Kimura H. (2002). Hydrogen sulfide as a neuromodulator. Mol. Neurobiol..

[6] Mikami Y., Shibuya N., Kimura Y., Nagahara N., Yamada M., Kimura H. (2011). Hydrogen sulfide protects the retina from light-induced degeneration by the modulation of Ca^2+^ influx. J. Biol. Chem..

[7] Shibuya N., Tanaka M., Yoshida M., Ogasawara Y., Togawa T., Ishii K., Kimura H. (2009). 3-Mercaptopyruvate sulfurtransferase produces hydrogen sulfide and bound sulfane sulfur in the brain. Antioxid. Redox Signal..

[8] Mikami Y., Shibuya N., Ogasawara Y., Kimura H. (2013). Hydrogen sulfide is produced by cystathionine γ-lyase at the steady-state low intracellular Ca^2+^ concentrations. Biochem. Biophys. Res. Commun..

[9] Beard M.E., Davies T., Holloway M., Holtzman E. (1988). Peroxisomes in pigment epithelium and Müller cells of amphibian retina possess D-amino acid oxidase as well as catalase. Exp. Eye Res..

[10] Koga R., Miyoshi Y., Sakaue H., Hamase K., Konno R. (2017). Mouse d-Amino-Acid Oxidase: Distribution and Physiological Substrates. Front. Mol. Biosci..

[11] Buffault J., Labbé A., Hamard P., Brignole-Baudouin F., Baudouin C. (2020). The trabecular meshwork: Structure, function and clinical implications. A review of the literature. J. Fr. D’ophtalmol..

[12] Stamer W.D., Acott T.S. (2012). Current understanding of conventional outflow dysfunction in glaucoma. Curr. Opin. Ophthalmol..

[13] Salvi A., Bankhele P., Jamil J.M., Kulkarni-Chitnis M., Njie-Mbye Y.F., Ohia S.E., Opere C.A. (2016). Pharmacological Actions of Hydrogen Sulfide Donors on Sympathetic Neurotransmission in the Bovine Anterior Uvea, In Vitro. Neurochem. Res..

[14] Robinson J., Okoro E., Ezuedu C., Bush L., Opere C.A., Ohia S.E., Njie-Mbye Y.F. (2017). Effects of Hydrogen Sulfide-Releasing Compounds on Aqueous Humor Outflow Facility in Porcine Ocular Anterior Segments, Ex Vivo. J. Ocul. Pharmacol. Ther. Off. J. Assoc. Ocul. Pharmacol. Ther..

[15] Huang S., Huang P., Liu X., Lin Z., Wang J., Xu S., Guo L., Leung C.K., Zhong Y. (2017). Relevant variations and neuroprotective effect of hydrogen sulfide in a rat glaucoma model. Neuroscience.

[16] Freedman J., Goddard D. (2008). Elevated levels of transforming growth factor beta and prostaglandin E2 in aqueous humor from patients undergoing filtration surgery for glaucoma. Can. J. Ophthalmol..

[17] Mermoud A., Baerveldt G., Minckler D.S., Rao N.A. (1995). Prostaglandines E2 et F2-alpha au cours du glaucome uvéitique chez le rat Lewis [Prostaglandins E2 and F2-alpha in uveitic glaucoma in the Lewis rat]. Klin. Monatsblatter Augenheilkd..

[18] Monjok E.M., Kulkarni K.H., Kouamou G., McKoy M., Opere C.A., Bongmba O.N., Njie Y.F., Ohia S.E. (2008). Inhibitory action of hydrogen sulfide on muscarinic receptor-induced contraction of isolated porcine irides. Exp. Eye Res..

[19] Ohia S.E., Opere C.A., Monjok E.M., Kouamou G., Leday A.M., Njie-Mbye Y.F. (2010). Role of hydrogen sulfide production in inhibitory action of L-cysteine on isolated porcine irides. Curr. Eye Res..

[20] Asimakopoulou A., Panopoulos P., Chasapis C.T., Coletta C., Zhou Z., Cirino G., Giannis A., Szabo C., Spyroulias G.A., Papapetropoulos A. (2013). Selectivity of commonly used pharmacological inhibitors for cystathionine β synthase (CBS) and cystathionine γ lyase (CSE). Br. J. Pharmacol..

[21] Porter D.W., Baskin S.I. (1996). The effect of three alpha-keto acids on 3-mercaptopyruvate sulfurtransferase activity. J. Biochem. Toxicol..

[22] Chitnis M.K., Njie-Mbye Y.F., Opere C.A., Wood M.E., Whiteman M., Ohia S.E. (2013). Pharmacological actions of the slow-release hydrogen sulfide donor GYY4137 on phenylephrine-induced tone in isolated bovine ciliary artery. Exp. Eye Res..

[23] Kulkarni-Chitnis M., Njie-Mbye Y.F., Mitchell L., Robinson J., Whiteman M., Wood M.E., Opere C.A., Ohia S.E. (2015). Inhibitory action of novel hydrogen sulfide donors on bovine isolated posterior ciliary arteries. Exp. Eye Res..

[24] Pong W.W., Stouracova R., Frank N., Kraus J.P., Eldred W.D. (2007). Comparative localization of cystathionine beta-synthase and cystathionine gamma-lyase in retina: Differences between amphibians and mammals. J. Comp. Neurol..

[25] Acott T.S., Kelley M.J., Keller K.E., Vranka J.A., Abu-Hassan D.W., Li X., Aga M., Bradley J.M. (2014). Intraocular pressure homeostasis: Maintaining balance in a high-pressure environment. J. Ocul. Pharmacol. Ther..

[26] Vranka J.A., Acott T.S. (2017). Pressure-induced expression changes in segmental flow regions of the human trabecular meshwork. Exp. Eye Res..

[27] Vranka J.A., Staverosky J.A., Raghunathan V., Acott T.S. (2020). Elevated pressure influences relative distribution of segmental regions of the trabecular meshwork. Exp. Eye Res..

[28] Schneemann A., Leusink-Muis A., van den Berg T., Hoyng P.F., Kamphuis W. (2003). Elevation of nitric oxide production in human trabecular meshwork by increased pressure. Graefes Arch. Clin. Exp..

[29] Kulkarni M., Njie-Mbye Y.F., Okpobiri I., Zhao M., Opere C.A., Ohia S.E. (2011). Endogenous production of hydrogen sulfide in isolated bovine eye. Neurochem. Res..

[30] Abe K., Kimura H. (1996). The possible role of hydrogen sulfide as an endogenous neuromodulator. J. Neurosci. Off. J. Soc. Neurosci..

[31] Kolluru G.K., Shen X., Bir S.C., Kevil C.G. (2013). Hydrogen sulfide chemical biology: Pathophysiological roles and detection. Nitric Oxide Biol. Chem..

[32] Huang K., Wang Z., Gu Y., Hu Y., Ji Z., Wang S., Lin Z., Li X., Xie Z., Pan S. (2016). Glibenclamide Is Comparable to Target Temperature Management in Improving Survival and Neurological Outcome After Asphyxial Cardiac Arrest in Rats. J. Am. Heart Assoc..

[33] Winkler N.S., Fautsch M.P. (2014). Effects of prostaglandin analogues on aqueous humor outflow pathways. J. Ocul. Pharmacol. Ther..

[34] Toris C.B., Gabelt B.T., Kaufman P.L. (2008). Update on the mechanism of action of topical prostaglandins for intraocular pressure reduction. Surv. Ophthalmol..

[35] Bahler C.K., Howell K.G., Hann C.R., Fautsch M.P., Johnson D.H. (2008). Prostaglandins increase trabecular meshwork outflow facility in cultured human anterior segments. Am. J. Ophthalmol..

[36] Maihöfner C., Schlötzer-Schrehardt U., Gühring H., Zeilhofer H.U., Naumann G.O., Pahl A., Mardin C., Tamm E.R., Brune K. (2001). Expression of cyclooxygenase-1 and -2 in normal and glaucomatous human eyes. Investig. Ophthalmol. Vis. Sci..

[37] Kwiatkoski M., Soriano R.N., Araujo R.M., Azevedo L.U., Batalhao M.E., Francescato H.D., Coimbra T.M., Carnio E.C., Branco L.G. (2013). Hydrogen sulfide inhibits preoptic prostaglandin E2 production during endotoxemia. Exp. Neurol..

[38] Sun Q., Chen Z., He P., Li Y., Ding X., Huang Y., Gu H., Ni X. (2018). Reduced Expression of Hydrogen Sulfide-Generating Enzymes Down-Regulates 15-Hydroxyprostaglandin Dehydrogenase in Chorion during Term and Preterm Labor. Am. J. Pathol..

[39] Ang S.F., Sio S.W., Moochhala S.M., MacAry P.A., Bhatia M. (2011). Hydrogen sulfide upregulates cyclooxygenase-2 and prostaglandin E metabolite in sepsis-evoked acute lung injury via transient receptor potential vanilloid type 1 channel activation. J. Immunol..

[40] Njie Y.F., Qiao Z., Xiao Z., Wang W., Song Z.H. (2008). N-arachidonylethanolamide-induced increase in aqueous humor outflow facility. Investig. Ophthalmol. Vis. Sci..

[41] Yang Y.F., Sun Y.Y., Acott T.S., Keller K.E. (2016). Effects of induction and inhibition of matrix cross-linking on remodeling of the aqueous outflow resistance by ocular trabecular meshwork cells. Sci. Rep..

[42] Smith H.M., Pluth M.D. (2023). Advances and Opportunities in H_2_S Measurement in Chemical Biology. JACS Au.

[43] Zou S., Shimizu T., Shimizu S., Higashi Y., Nakamura K., Ono H., Aratake T., Saito M. (2018). Possible role of hydrogen sulfide as an endogenous relaxation factor in the rat bladder and prostate. Neurourol. Urodyn..

[44] Kruger N.J. (1994). The Bradford method for protein quantitation. Methods Mol. Biol..

